# Role of EIF4G1 network in non‐small cell lung cancers (NSCLC) cell survival and disease progression

**DOI:** 10.1111/jcmm.16307

**Published:** 2021-02-04

**Authors:** Luis Del Valle, Lu Dai, Hui‐Yi Lin, Zhen Lin, Jungang Chen, Steven R. Post, Zhiqiang Qin

**Affiliations:** ^1^ Department of Pathology Louisiana State University Health Sciences Center Louisiana Cancer Research Center New Orleans LA USA; ^2^ Department of Pathology Winthrop P. Rockefeller Cancer Institute University of Arkansas for Medical Sciences Little Rock AR USA; ^3^ Biostatistics Program School of Public Health Louisiana State University Health Sciences Center New Orleans LA USA; ^4^ Department of Pathology Tulane University Health Sciences Center Tulane Cancer Center New Orleans LA USA

**Keywords:** EIF4G1, lung cancer, MUC1, NRG1, NSCLC

## Abstract

Although the Eukaryotic Translation Initiation Factor 4 Gamma 1 (EIF4G1) has been found overexpressed in a variety of cancers, its role in non–small cell lung cancers (NSCLC) pathogenesis especially in immunoregulatory functions, its clinical relevance and therapeutic potential remain largely unknown. By using cancer patients tissue assays, the results indicate that EIF4G1 expressional levels are much higher in NSCLC tissues than in adjacent or normal lung tissues, which are also associated with NSCLC patient survival. By using an RNA‐Sequencing based pipeline, the data show that EIF4G1 has a significant association with immune checkpoint molecules such as PD‐1/PD‐L1 in NSCLC. EIF4G1 small‐molecule inhibitors effectively repress NSCLC growth in cell culture and xenograft animal models. Protein array results identify the signature of proteins controlled by EIF4G1 in NSCLC cells, in which new candidates such as MUC1 and NRG1 are required for NSCLC survival and tumorigenesis with clinical relevance. Taken together, these results have for the first time demonstrated the immunoregulatory functions, clinical relevance and therapeutic potential of the EIF4G1 network in NSCLC, which may represent a promising and novel target to improve lung cancer treatment.

## 1 INTRODUCTION

Lung cancer is the number one killer among cancers in the United States with an estimated 154,050 deaths reported in 2018.^1^ It is also the second most diagnosed cancer in the United States and is responsible for more than 200,000 new cases per year.^1^ Lung cancers can be broadly classified as small cell lung cancer (SCLC) and non–small cell lung cancer (NSCLC) with NSCLCs accounting for about 85%‐90% of lung cancer cases.^2^ Clinically, the majority of lung cancer patients do not respond well to current chemo‐/radiotherapeutic regimens and have a dismal 5‐year survival rate of less than 15%.^3^ Recently, the introduction of targeted therapy and immunotherapy has given new hope to NSCLC patients, but the outcome/prognosis is still far from satisfactory. Immunotherapy more recently has become the most revolutionary treatment for solid tumours patients. Among NSCLC patients with PD‐L1 expression in more than 50% of tumour cells, treatment with Pembrolizumab, which targets PD‐1, leads to a superior progression‐free and overall survival compared to platinum‐doublet chemotherapy in the first‐line setting.^4^ Furthermore, the addition of Pembrolizumab to standard chemotherapy of pemetrexed and a platinum‐based drug resulted in significant longer progression‐free survival and overall survival irrespective to PD‐L1 expression.^5^ However, the resistance to immunotherapy and hyper‐progressive disease of checkpoint inhibitors treatment has been recently reported in some NSCLC patients.^6^, ^7^ Thus, there is still an urgent need to better understand the mechanisms of lung carcinogenesis and to develop novel therapies (alone or combination of existing treatments) for lung cancer patients.

Dysregulation of mRNA translation is a frequent feature of neoplasia. Therefore, therapeutic agents that target components of the protein synthesis apparatus hold promise as novel anticancer drugs that can overcome intra‐tumour heterogeneity.^8^ Over the last two decades, the eukaryotic initiation factor 4F (EIF4F) complex has been shown to play important roles in oncogenesis.^9^, ^10^ As an important component of the EIF4F complex, EIF4G1 protein serves as a scaffold and interacts with several other initiation factors such as EIF4E and EIF4A and helps to initiate cap‐dependent translation in mammalian cells by recruiting ribosomes to the capped end of mRNA.^11^ In contrast to extensive studies of EIF4E and EIF4A components, much less attention has been paid to the function of EIF4G1. For example, several recent studies have indicated that EIF4E protein levels are associated with NSCLC cell proliferation, migration, invasion, epithelial‐to‐mesenchymal transition (EMT) and chemo‐resistance.^12^, ^13^, ^14^ Although EIF4G1 is overexpressed in a variety of cancers, its role in NSCLC pathogenesis especially immunoregulatory functions, clinical relevance and therapeutic potential remains largely unknown. One study showed that 4EGI‐1, one of EIF4G1 inhibitors, enhanced the apoptotic effects of tumour necrosis factor‐related apoptosis‐inducing ligand (TRAIL) on NSCLC cell lines, through inducing CCAAT/enhancer binding protein homologous protein‐dependent DR5 and ubiquitin/proteasome‐mediated degradation of cellular FLICE‐inhibitory protein (c‐FLIP).^15^ However, the investigators do not focus on the cellular functions of EIF4G1 including its controlled downstream proteins, and the efficacy of EIF4G1 targeted therapy in vivo remains unclear.

Our recently published data demonstrate for the first time that stable silencing of *EIF4G1* by shRNA causes significant reduction of proliferation and anchorage‐independent growth in NSCLC cell lines (eg A549, H460, H1299). Furthermore, EIF4G1 was potentially required for NSCLC metastasis through promoting tumour cell migration and invasion.^16^, ^17^ In the current study, we explore the clinical implications of EIF4G1 by using NSCLC tissue microarrays and other clinical databases, determine the efficacy of selective EIF4G1 inhibitors in NSCLC xenograft models and identify new EIF4G1‐controlled cellular proteins in NSCLC cells as well as validating their functions.

## 2 MATERIALS AND METHODS

### 2.1 Cell culture and reagents

NSCLC cell lines (A549, H460, H1299) were kindly provided by Dr Hua Lu at Tulane University (purchased from ATCC, Manassas, VA, USA) and cultured in RPMI‐1640 medium supplemented with 10% foetal bovine serum and 1% penicillin and streptomycin. All experiments were performed with mycoplasma‐free cells. 4EGI‐1 and 4E1RCat were purchased from SelleckChem (Houston, TX, USA). NSCLC formalin‐fixed, paraffin‐embedded (FFPE) tissue arrays were purchased from US Biomax (Derwood, MD, USA), containing normal lung tissues (n = 10), tumour and paired adjacent tissues from the same patients (n = 45), including 19 cases of LUAD (lung adenocarcinomas) and 26 cases of LUSC (lung squamous cell carcinomas). Protein arrays and analyses were performed in RayBiotech, Inc (Guangzhou, China) by using RayBio Human Protein Array G (Glass–chip‐based protein arrays containing 499 targets).

### 2.2 Immunohistochemistry

Immunohistochemistry was performed with the Avidin‐Biotin‐Peroxidase complex, according to the manufacturer's instructions (Vector Laboratories). Our modified protocol includes paraffin melting at 58°C in a regular oven for 20 minutes, deparaffination in xylene, re‐hydration through descending grades of alcohol up to water, and non‐enzymatic antigen retrieval in 0.01 M sodium citrate buffer, pH 6.0, heated to 95°C for 40 minutes in a vacuum oven. After a cooling period of 30 minutes, the slides were rinsed in PBS and treated with 3% H_2_O_2_ in methanol for 25 minutes to quench endogenous peroxidase. Sections were then blocked with 5% normal horse serum (for mouse monoclonal antibodies), or normal goat serum (for rabbit polyclonal antibodies) in 0.1% PBS/BSA for 2 hours at room temperature. Primary antibodies were incubated overnight at room temperature in a humidifier chamber. Primary antibodies utilized in the present study included rabbit polyclonal anti‐EIF4G1 (Abcam, Cambridge, MA, USA, Cat. #ab2609, 1:100 dilution), anti‐MUC‐1/CA15‐3 (Proteintech, Chicago, IL, USA, Cat. # 19976‐1‐AP, 1:100 dilution) and anti‐NRG1 (Abcam, Cat. #ab53104, 1:500 dilution), a mouse monoclonal anti‐PD‐1 (Abcam, Cat. #ab52587, 1:100 dilution) and a rabbit monoclonal anti‐PD‐L1 (Abcam, Cat. #ab205921, 1:500 dilution). In the second day, slides were thoroughly rinsed with PBS, and biotinylated secondary anti‐mouse or anti‐rabbit antibodies were incubated for 1 hour at room temperature (1:200 dilution). Then, sections were incubated with avidin‐biotin‐peroxidase complexes (Vectastain ABC Elite kit; Vector Laboratories) for 1 hour at room temperature, rinsed with PBS and developed with diaminobenzidine (DAB tablets, Sigma, St. Louis, MO, USA) for three minutes. Finally, the sections were counterstained with Haematoxylin and mounted with Permount (Fisher Scientific, Waltham, MA, USA).

### 2.3 Cell proliferation assays

Cell proliferation was measured using the WST‐1 assay (Roche, Indianapolis, IN, USA). Briefly, after the period of treatment of cells, 10 μL/well of cell proliferation reagent, WST‐1 (4‐[3‐(4‐Iodophenyl)‐2‐(4‐nitro‐ phenyl)‐2H‐5‐tetrazolio]‐1,3‐benzene disulfonate), was added into 96‐well microplate and incubated for 3 h at 37°C in 5% CO_2_. The absorbance of samples was measured by using a microplate reader at 490 nm.

### 2.4 Immunoblotting

Total cell lysates (20μg) were resolved by 10% SDS–PAGE, transferred to nitrocellulose membranes and immunoblotted with antibodies for EIF4G1, NRG1 (Abcam), MUC1 (Proteintech), phosphor‐ and total‐p65 (Cell Signaling, Danvers, MA, USA, Cat. #3033 and #4764, respectively) and β‐Actin (Sigma‐Aldrich, Cat. #SAB5500001) for loading controls. Immunoreactive bands were identified using an enhanced chemiluminescence reaction (Perkin‐Elmer, Waltham, MA, USA) and visualized by autoradiography.

### 2.5 RNA interference assays

For RNA interference (RNAi) assays, *EIF4G1, MUC1 and NRG1* On‐Target plus SMART pool small interfering RNA (siRNA; Dharmacon, Lafayette, CO, USA) or negative control siRNA, were delivered using the DharmaFECT Transfection Reagent as recommended by the manufacturer. To establish stably *EIF4G1*, *MUC1* and/or *NRG1* knockdown cells, we used Dharmacon lentiviral vectors containing 2 shRNA specifically for each targeted gene (KD1 and KD2) and a non‐silencing control (NC)‐shRNA.

### 2.6 NSCLC xenograft models

Cells were counted and washed once in ice‐cold PBS. 5x10^5^ H460 cells in 50 µL PBS plus 50 µL growth factor‐depleted Matrigel (BD Biosciences) were injected subcutaneously into the flank of nude mice, 6‐8‐week old, male/female (Jackson Laboratory). Two days after this injection, the mice were randomly separated into different groups (4‐6 mice per group) and received i.p. injection with either vehicle, 4EGI‐1 or 4E1RCat (25 mg/kg of bodyweight, respectively), 3 days/week. The mice were observed and measured every 3 days for the size of palpable tumours for additional 3 weeks. At the end of experiment, the tumours were excised for subsequent histopathological analysis. Haematoxylin & Eosin (H&E) and immunohistochemistry (IHC) for Ki67 were performed as described previously.^18^, ^19^ Images were collected using an Olympus BX61 microscope equipped with a high‐resolution DP72 camera and CellSense image capture software. To test the roles of MUC1 and NRG1 in NSCLC tumorigenesis, 5x10^5^ H460 stably *MUC1*‐shRNA, *NRG1*‐shRNA knockdown cells or NC‐shRNA cells were injected subcutaneously into nude mice, respectively. After 4 weeks, the mice were killed and tumours were excised and compared. All the animal protocols were approved (# 3380) by the LSUHSC Animal Care and Use Committees in accordance with national guidelines.

### 2.7 Statistical analysis

The protein expression of EIF4G1, PD‐1, PD‐L1, MUC1 and NRG1 in tumour tissues was measured as an ordinal variable with the four strength levels: negative (‐), weak (+), intermediate (++) and strong (+++/++++). The pairwise correlations of proteins in NSCLC tumour tissues were tested using the Spearman correlations. The correlations of protein expression levels in NSCLC tumour tissues were evaluated using the Pearson correlations. Differences of protein expression levels between experimental and control groups were tested using the two‐sided t test, and *P* values < .05 were considered significant. The 50% Inhibitory Concentrations (IC_50_) were calculated by using SPSS v20.0.

## 3 RESULTS

### 3.1 Clinical implications of EIF4G1 in NSCLC patients

**FIGURE 1:**
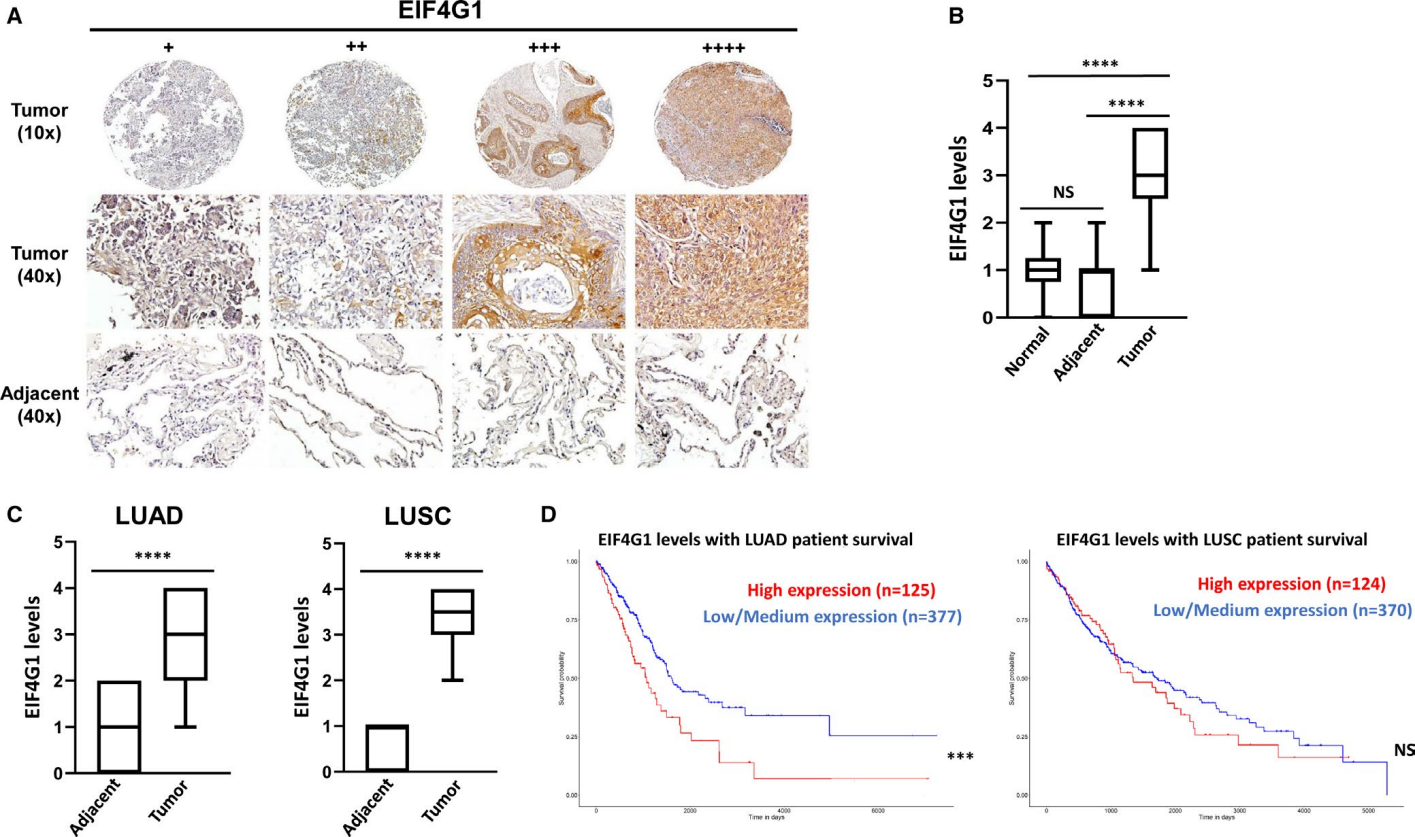
Elevation of EIF4G1 expression in NSCLC tumour tissues. Expression of EIF4G1 in formalin‐fixed paraffin‐embedded (FFPE) NSCLC tissue arrays, which contain normal lung tissues (n = 10), tumour and paired adjacent tissues from the same patients (n = 45), was determined by immunohistochemistry. A, The IHC images from representative cases, +: weak; ++: intermediate; +++/++++: robust. B‐C, The Box plots show expressional difference among these groups, LUAD (lung adenocarcinoma, n = 19); LUSC (lung squamous carcinoma, n = 26). D, Patient survival data from the TCGA NSCLC cohort were used for Kaplan‐Meier survival analyses to create the overall survival plots. High expression: gene TPM (transcript per million) values above upper quartile. Low/Medium expression: gene TPM values below upper quartile. The survival curves of samples with high or low/medium gene expression were compared by log‐rank test. The p value obtained from log‐rank test was used to measure the statistical significance of survival correlation. *** = *P* < .001; **** = *P* < .0001; NS: no significant

NSCLC tissue arrays, which contain normal lung tissues (n = 10), tumour and paired adjacent tissues from the same patients (n = 45), were used to measure EIF4G1 expression through immunohistochemistry. The results indicated that in almost all of patients (LUAD and LUSC), EIF4G1 expression was significantly more robust in tumour tissues than paired adjacent or normal lung tissues (Figure 1A‐C), although its expressional levels showed some variation among different patients’ tumour tissues based on IHC scores: + (2/45), ++ (9/45), +++ (12/45), ++++ (22/45). The information about clinical characters and IHC scores for each patient involved in tissue arrays were listed in Supplemental Table 1. However, no significant correlation was found between EIF4G1 expression and tumour TNM, grade or stage (data not shown), which is probably because of the limited number of cases analysed here. By using NSCLC clinical data from The Cancer Genome Atlas (TCGA) cohort, we found that the mRNA level of *EIF4G1* were closely related to NSCLC patients’ overall survival through Kaplan‐Meier survival analyses, particularly in LUAD patients (Figure 1D). Taken together, these data strongly support the important clinical relevance of EIF4G1 in NSCLC progression and pathogenesis.

**TABLE 1:** The top 10 proteins significantly up‐regulated and/or down‐regulated in NSCLC H1299 *EIF4G1* stably knockdown cell line identified from protein array analyses

Gene symbol	Protein description	Fold change
		H1299 *EIF4G1^‐^*/H1299 control
*ALB*	Albumin	29.1
*AGT*	Angiotensinogen	26.9
*NTF3*	Neurotrophin 3	19.7
*TGFB3*	Transforming growth factor, beta 3	9.7
*CA242*	Cancer Antigen 242	8.5
*ANGPT4*	Angiopoietin 4	7.0
*NTN1*	Netrin 1	6.3
*NOV*	Nephroblastoma overexpressed	4.4
*CA27‐29*	Cancer Antigen 27‐29	3.4
*CCL19*	Chemokine (C‐C motif) ligand 19	3.4
*CCR5*	Chemokine (C‐C motif) receptor 5 (gene/pseudogene)	0.2
*CCL14*	Chemokine (C‐C motif) ligand 14	0.15
*GAPDH*	Glyceraldehyde‐3‐phosphate dehydrogenase	0.14
*SDC1*	Syndecan 1	0.13
*MUC1*	Mucin 1, cell surface associated	0.09
*PPY*	Pancreatic polypeptide	0.06
*PGF*	Placental growth factor	0.06
*PGLYRP1*	Peptidoglycan recognition protein 1	0.04
*CXCL3*	Chemokine (C‐X‐C motif) ligand 3	0.04
*NRG1*	Neuregulin 1	0.03

### 3.2 Regulation of immune checkpoint molecules by EIF4G1 in NSCLC patients

**FIGURE 2:**
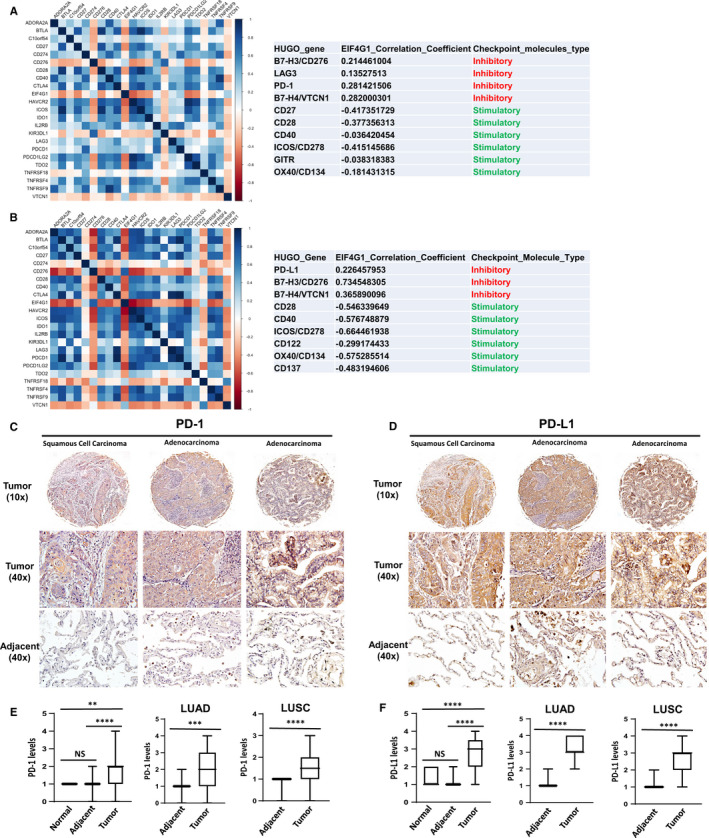
Correlation of EIF4G1 and common immune checkpoint molecules in NSCLC biopsies. (A‐B) RNA‐Sequencing data sets of 20 LUAD (A) and 20 LUSC (B) samples were obtained from TCGA cohort. Gene expression was determined by the RSEM pipeline using GRCH38 reference genome as previously described. The Pearson correlations were calculated based on the gene TPM values and visualized as the heat maps using R packages. (C‐D) IHC images of PD‐1 (C) and PD‐L1 (D) labelling from representative cases of NSCLC tissue arrays. (E‐F) The Box plots show expressional differences among these groups, including normal lung tissues (n = 10), tumour and paired adjacent tissues (n = 45 containing 19 cases of LUAD and 26 cases of LUSC). ** = *P* < .01; *** = *P* < .001; **** = *P* < .0001; NS: no significant

Recent studies have revealed that the EIF4F complex may have immunoregulatory functions (especially regulation of key immune checkpoint molecules) in the tumour microenvironment. For example, programmed cell death 1 (PD‐1) can bind to EIF4E and promote its phosphorylation in hepatocellular carcinoma cells.^20^ We recently have developed a RNA‐Sequencing‐based pipeline to analyse the correlation of key regulatory factors and common immune checkpoint molecules in tumour microenvironment. Gene expression was determined by RSEM pipeline using GRCH38 reference genome. The Pearson correlations were calculated based on the gene TPM values and visualized as the heat maps using R packages. After analysis of RNA‐Sequencing data sets of 20 LUAD and 20 LUSC samples from TCGA cohort, the results showed that EIF4G1 potentially up‐regulated inhibitory checkpoint molecules whereas down‐regulating stimulatory checkpoint molecules (Figure 2A‐B), which may facilitate tumour cell immune escape for both subtypes of NSCLC. To further confirm these analyses, the same NSCLC tissue arrays above were used to detect the expressional levels of PD‐1 and PD‐L1, two of major immune checkpoint molecules. The results indicated that both PD‐1 and PD‐L1 expressions were significantly higher in tumour tissues than paired adjacent or normal lung tissues (Figure 2C‐F). Moreover, most of NSCLC cases with positive for EIF4G1 expression (++ ~ ++++) were also positive for PD‐1 (53.3%, 24/45) and/or PD‐L1 (91.1%, 41/45). However, silencing of *EIF4G1* by RNAi was not able to down‐regulate PD‐L1 expression from NSCLC cell lines (Figure S1), indicating that EIF4G1 does not directly control PD‐L1 translation.

### 3.3 EIF4G1 selective inhibitors effectively repress NSCLC cell growth

**FIGURE 3:**
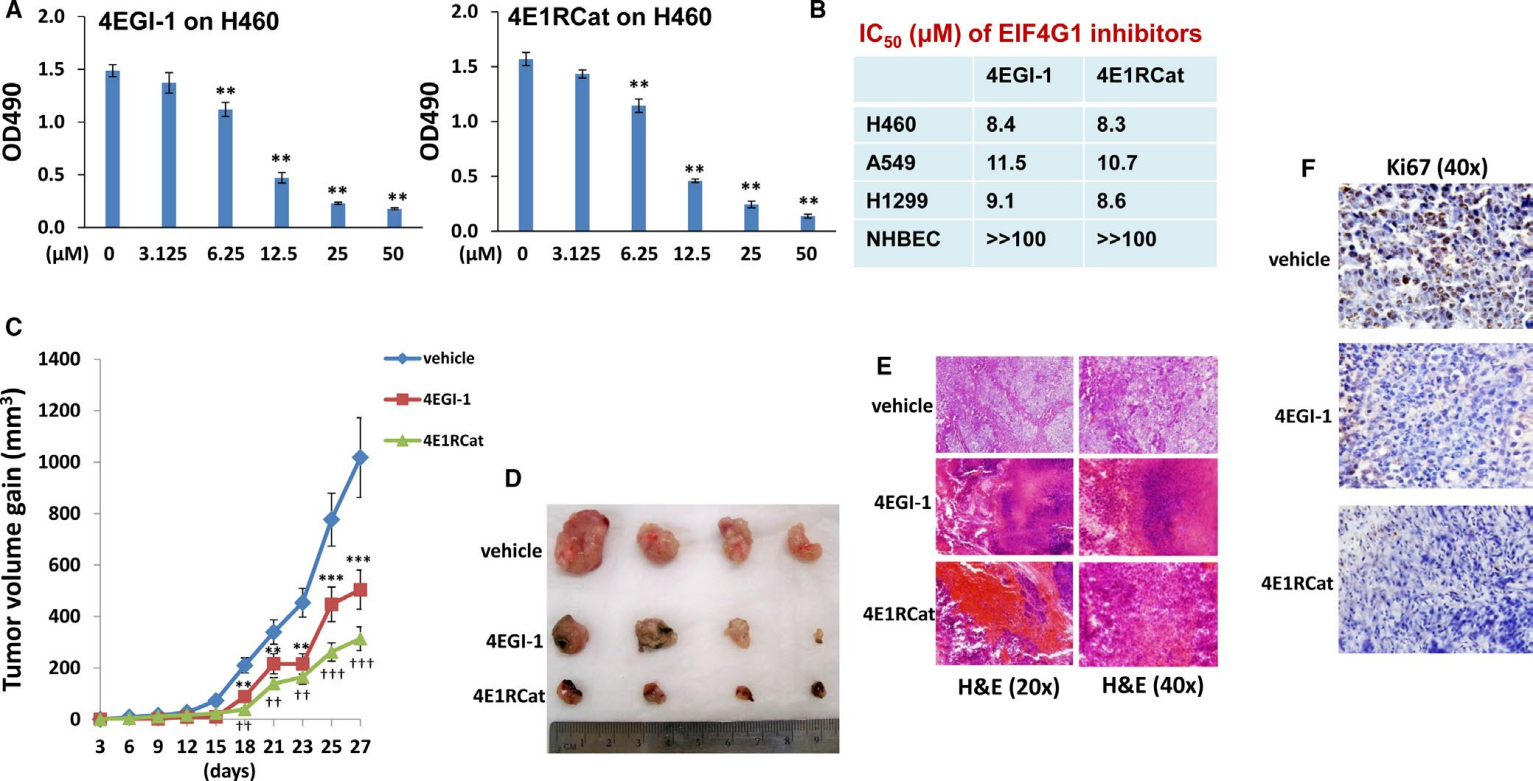
Targeting EIF4G1 by selective inhibitors represses NSCLC tumour growth. (A‐B) NSCLC cell lines (H460, A549, H1299) or normal human bronchial epithelial cells (NHBEC) were incubated with the indicated concentrations of EIF4G1 inhibitors (4EGI‐1 or 4E1RCat) for 48 hours. The cell proliferation was examined using the WST‐1 cell proliferation assays (Roche). Error bars represent the SD for 3 independent experiments. The 50% Inhibitory Concentration (IC_50_) was calculated by using SPSS 20.0 software. (C‐D) NSCLC cells H460 (5x10^5^ cells 1:1 with growth factor‐depleted Matrigel) were injected subcutaneously into nude mice. After 48 hours, mice were received i.p. injection with either vehicle, 4EGI‐1 or 4E1RCat (25 mg/kg of bodyweight, respectively), 3 days/week. The mice were observed and measured every 3 days for the size of palpable tumours for additional 3 weeks. At the end of experiment, the tumours were excised for subsequent analysis. Error bars represent SD for different mice in the same group. **/†† = *P* < .01; ***/††† = *P* < .001. (E‐F) H&E and Ki67 labelling shows that 4EGI‐1 and/or 4E1RCat treatments caused extensive necrosis within tumour tissues and significant inhibition of tumour cell proliferation

Currently, there are a limited number of EIF4G1 specific inhibitors commercially available. 4EGI‐1 and 4E1RCat are two small‐molecule competitive inhibitors that can prevent EIF4G:EIF4E interaction as well as EIF4F complex formation.^8^ Our results indicated that both 4EGI‐1 and 4E1RCat treatments effectively reduced the growth of NSCLC cell lines, including H460, A549, H1299, in a dose‐dependent manner (IC_50_ at ~ 8.0‐11.0 μM), whereas they displayed almost no inhibitory effects on normal human bronchial epithelial cells (NHBEC) at the same range of concentrations (IC_50_ » 100 μM) (Figure 3A‐B). This is probably because of the obvious elevation of EIF4G1 expression in NSCLC cell lines compared to normal lung cells as described previously.^16^ By using an established NSCLC xenograft model,^21^ the results indicated that both 4EGI‐1 and 4E1RCat treatments significantly repressed H460 tumour growth in vivo (Figure 3C‐D). H&E and Ki67 labelling revealed that these EIF4G1 inhibitors caused extensive necrosis within NSCLC tumour tissues and the obvious inhibition of tumour cell proliferation, when compared to the vehicle treatment controls (Figure 3E‐F).

### 3.4 Identification of the signature of EIF4G1‐controlled proteins in NSCLC cells

**FIGURE 4:**
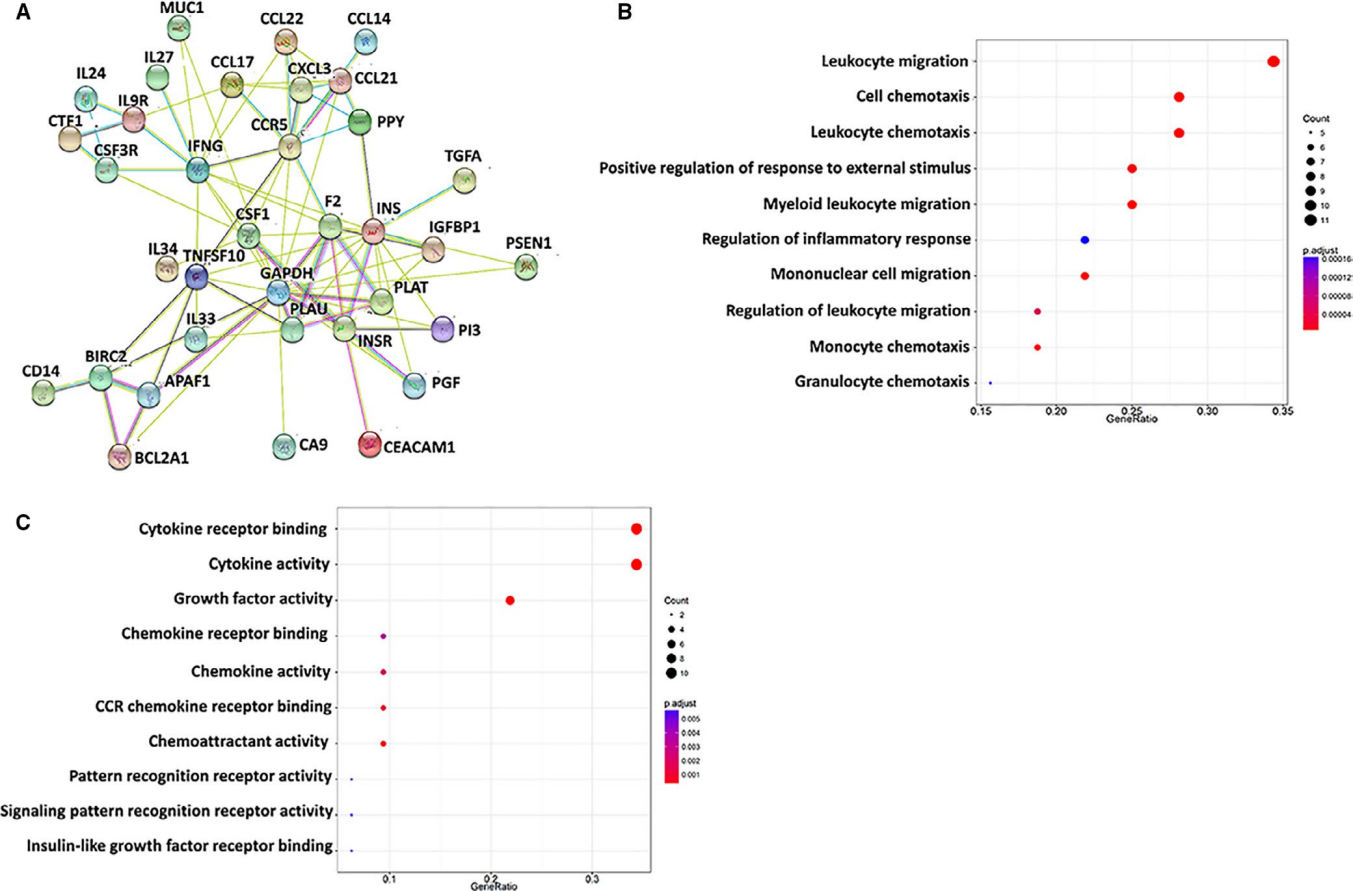
Signature of EIF4G1‐controlled protein expression in NSCLC cells. A, The protein profile between H1299 stably *EIF4G1*‐shRNA knockdown cell line and control shRNA cell line were compared using RayBio^®^ Human Protein Array. Protein‐protein interaction analysis was performed at https://string‐db.org/. The representation of action types and node colours has been listed on the website. B‐C, The enrichment analysis of protein profile was conducted using the Gene Ontology (GO) Biological Process (B) and Molecular Function (C) Modules of clusterProfiler, derived from R/bioconductor

As the principal function of EIF4G1 is to control cellular gene translation, a protein array was used for the identification of the signature EIF4G1‐controlled proteins in NSCLC cells. The results identified 24 proteins significantly up‐regulated and 54 proteins significantly down‐regulated (≥ twofold & *P* < .05) from *EIF4G1* stably knockdown H1299 cell line when compared to the parental cell line. The top 10 significantly up‐regulated or down‐regulated proteins were listed in Table 1. Most of these downstream proteins have never been reported to be regulated by EIF4G1, whereas they showed a close protein‐protein interaction network and potential functional association (Figure 4A). Interestingly, the Gene Ontology (GO) enrichment analysis indicated that many of EIF4G1‐controlled proteins were involved in immune cell migration, chemotaxis, regulation of inflammatory responses, including cytokines and chemokines activities (Figure 4B‐C). KEGG (Kyoto Encyclopedia of Genes and Genomes) pathway analysis indicated that the NF‐κB signalling pathway activity was potentially affected by EIF4G1 (Figure S2A). The immunoblot results confirmed that knockdown of *EIF4G1* by RNAi significantly reduced the phosphorylation of NF‐κB p65 kinase from all the 3 NSCLC cell lines being tested (Fig. S2B). Notably, some recent studies have reported the regulation of immune checkpoint molecules by NF‐κB signalling pathway in different types of cancer.^22^, ^23^, ^24^ Taken together, these data indicate again the potential immunoregulatory function of EIF4G1 network in NSCLC tumour cells.

### 3.5 Novel EIF4G1‐controlled cellular proteins, MUC1 and NRG1, functional validation and clinical implications in NSCLC

**FIGURE 5:**
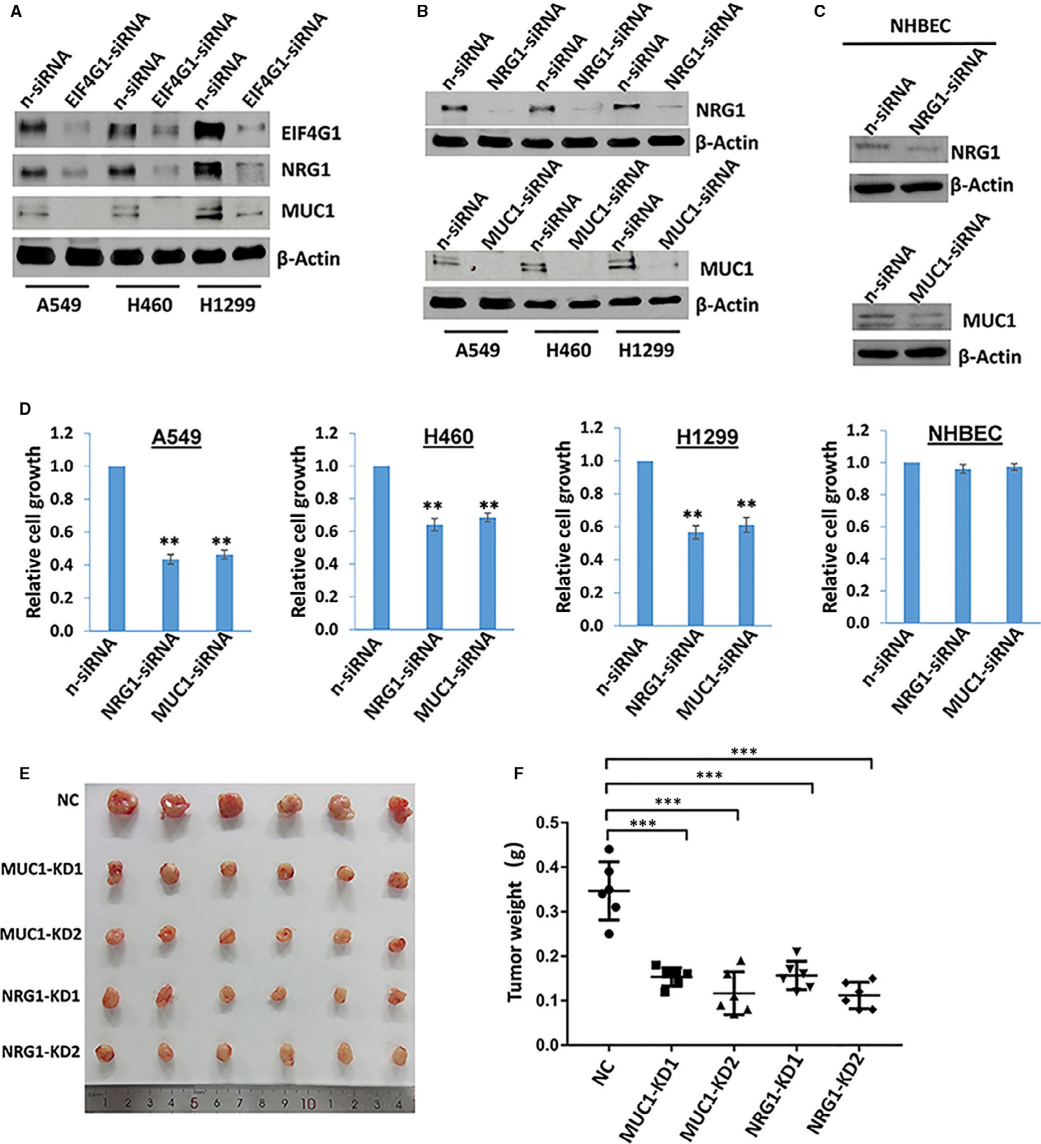
Direct targeting EIF4G1‐controlled proteins inhibits NSCLC cell growth in vitro and in vivo. A‐C, NSCLC cell lines (H460, A549, H1299) or normal human bronchial epithelial cells (NHBEC) were transfected with *EIF4G1*‐siRNA, *NRG1*‐siRNA, *MUC1*‐siRNA or non‐target control siRNA (n‐siRNA) for 72 hours; then, protein expression was measured by immunoblots. Representative blots from one of two independent experiments were shown. D, The cells were transfected as above; then, cell proliferation was examined using the WST‐1 assays (Roche). Error bars represent the SD for 3 independent experiments. ** = *P* < .01. (E‐F) The H460 stably *MUC1*‐shRNA, *NRG1*‐shRNA knockdown cell lines (each with lentiviral vectors containing 2 shRNA specifically targeting: MUC1‐ or NRG1‐KD1 and KD2, respectively) or control shRNA cell line were injected subcutaneously into nude mice. After 4 weeks, the mice were killed and tumours were excised and compared. Error bars represent SD for different mice in the same group. *** = *P* < .001

To further confirm protein array results with functional validation, two of EIF4G1‐controlled proteins newly identified, MUC1 (Mucin 1) and NRG1 (Neuregulin 1), were selected for subsequent investigation. MUC1 is a transmembrane glycoprotein that is aberrantly overexpressed in > 80% of NSCLC.^25^ Moreover, the overexpression of MUC1 in NSCLC is associated with a poor disease‐free and overall survival.^26^, ^27^ Another EIF4G1‐controlled protein, NRG1, is a ligand for the HER3 and HER4 receptors. NRG1 autocrine signalling has been implicated in insensitivity of NSCLC to EGFR inhibitors.^28^ Inhibition of NRG1‐ and other ligand‐mediated HER4 signalling can consistently and significantly enhance the response to chemotherapy and delay tumour regrowth after cessation of treatment.^29^ Our results first confirmed that silencing of *EIF4G1* by siRNA dramatically reduced MUC1 and NRG1 expression from all the 3 NSCLC cell lines (Figure 5A), which reflecting the accuracy of protein array data and the cell line relevance of these findings. In addition, directly silencing of *MUC1* or *NRG1* by siRNA significantly inhibited NSCLC cell growth, while almost not affecting NHBEC growth, which is probably because of the low basal levels of these proteins in normal lung cells (Figure 5B‐D). To further study the role of MUC1 and NRG1 in NSCLC tumorigenesis, the *MUC1* or *NRG1* stably knockdown H460 cell lines were created by using lentiviral vectors containing 2 shRNA specifically targeting each gene (KD1 and KD2, Figure S3). After being subcutaneously injected these cells or non‐targeting control (NC) cells into nude mice, the results showed that direct knockdown of *MUC1* or *NRG1* significantly repressed NSCLC tumour growth in mice (Figure 5E‐F).

**FIGURE 6:**
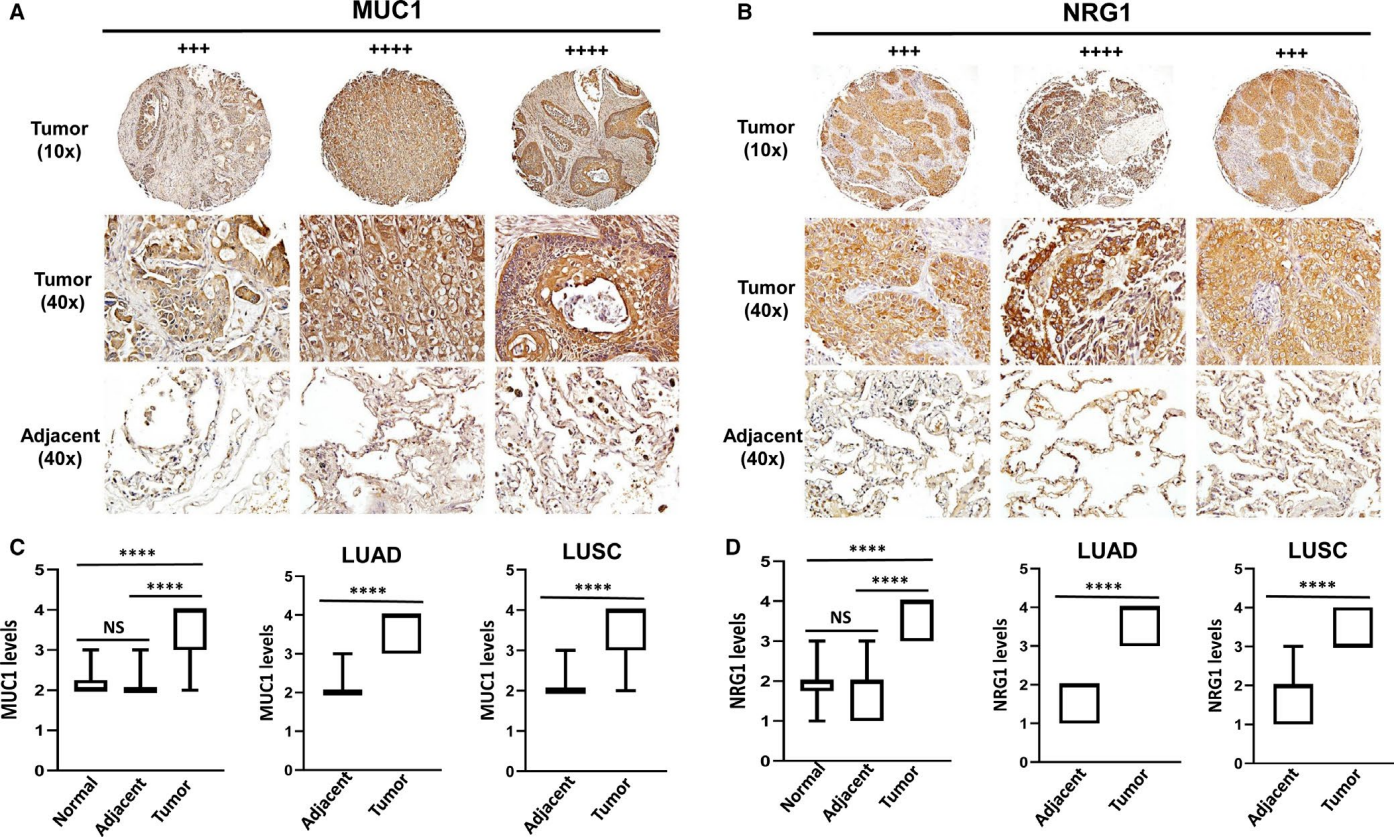
Elevation of MUC1 and NRG1 expression and their correlation with EIF4G1 in NSCLC tumour tissues. A‐B, Immunohistochemical images of MUC1 (A) and NRG1 (B) from representative cases of NSCLC tissue arrays. (C‐D) The box plots show expressional difference among these groups, including normal lung tissues (n = 10), tumour and paired adjacent tissues (n = 45 containing 19 cases of LUAD and 26 cases of LUSC). **** = *P* < .0001; NS: no significant

Next, the same NSCLC tissue arrays were used to detect the expressional levels of MUC1 and NRG1. The expression of both proteins was significantly higher in tumour tissues than paired adjacent or normal lung tissues (Figure 6A‐D). The Pearson correlations analysis indicated that EIF4G1 was positively correlated with NRG1 (*r* = 0.35; *P* = .01) rather than with MUC1 (*r* = 0.23; *P* = .12); interestingly, MUC1 and NRG1 displayed the strongest correlation in NSCLC (*r* = 0.43; *P* = .003). However, a higher number of NSCLC cases are needed for such analysis in future studies.

## 4 DISCUSSION

Our previous study reported that EIF4G1 was significantly up‐regulated in NSCLC cell lines compared to normal lung cells, which makes these lung cancer cells more susceptible to EIF4G1‐targeted therapy.^16^, ^17^ In the current study, we further demonstrate that EIF4G1 has much higher expressional levels in NSCLC tumour tissues than paired adjacent or normal lung tissues by using tissue microarrays, regardless of EIF4G1 IHC labelling scores for each case. The solid evidence has been provided that EIF4G1 inhibitors (eg 4EGI‐1 and 4E1RCat) display effective inhibition of NSCLC cell lines growth and tumorigenesis in vitro and in vivo. In contrast, these compounds show much less inhibitory effects on normal lung cells in the same doses range, because of the significant elevation of EIF4G1 and related protein levels in tumour cells. However, there are currently limited EIF4G1 specific inhibitors available. In a recent study, high‐throughput drug screening identified SBI‐0640756 as a new first‐in‐class inhibitor that targets EIF4G1 through disrupting the EIF4F complex and attenuates the growth of clinically unresponsive melanomas.^30^ Another recent study reported the design, synthesis, and in vitro characterization of a series of rigidified mimetic of 4EGI‐1 in which the phenyl in the 2‐(4‐(3,4‐dichlorophenyl)thiazol‐2‐yl) moiety was bridged into a tricyclic system.^31^ They found some analogues in this series to be markedly more potent than the parent prototypic inhibitor in the disruption of EIF4E/EIF4G interaction, and inhibition of human cancer cell proliferation. Therefore, the efficacy of these new compounds will be tested and compared for NSCLC once they are commercially available.

As mentioned before, recent immunotherapy studies have shown promising results on some NSCLC patients, despite of many existing challenges. One of key questions is how to improve immunotherapy efficacy through combination with other therapies including targeted therapy. In fact, EIF4E has been looked on as a node for regulation of immune functions via different translational control pathways,^32^ whereas much less known about EIF4G1 in this part. Here, we found that EIF4G1 has close associations with some immune checkpoint molecules such as PD‐1 and PD‐L1 in NSCLC by using RNA‐Sequencing and tissue arrays data from cancer patients. However, a lower positive labelling for PD‐1 than PD‐L1 was noticed in NSCLC tissues (Supplemental Table 1). Interestingly, one recent study reports that immunoreactivity loss driven by humidity and temperature results in structural distortion of epitopes rendering them unsuitable for antibody binding following epitope retrieval, especially during PD‐L1 immunohistochemistry in FFPE tissues.^33^ It remains unclear whether PD‐1 staining may have similar problems. To seek treatment benefits, we will explore whether EIF4G1 targeted therapy may improve immunotherapy (eg Pembrolizumab) in a new humanized NSCLC‐PDX (Patient‐derived xenograft) mice model recently established from the Jackson Laboratory.^34^

Although the EIF4F complex may control cellular protein translation, the protein array analyses indicated that only a limited number of proteins were significantly changed within *EIF4G1* stably knockdown H1299 cell line when compared to the controls, which is partially because of the detectable capability of protein array (~500 targets), even though these data imply the existence of a unique protein signature controlled by EIF4G1 in NSCLC cells. To prove that, we are currently working on getting other *EIF4G1* stably knockdown NSCLC cell lines and normal lung cell lines by shRNA for protein array analyses. CRISPR (clustered regularly interspaced short palindromic repeats)/ Cas9 (CRISPR‐associated gene 9) system ^35^, ^36^ is another option for gene editing, although completely knockout of *EIF4G1* is probably difficult to be achieved because of its essential function. In this study, two significantly down‐regulated proteins, MUC1 and NRG1, were selected from the candidate list of protein array results for functional validation. These data first confirmed that silencing of *EIF4G1* by RNAi dramatically reduced the expression of both proteins in multiple NSCLC cell lines, which reflects the accuracy of protein array data and the cell line relevance. We actually also observed that knockdown of *EIF4G1* partially reduced mRNA levels of *MUC1* and *NRG1* (data not shown), although the underlying mechanisms still require investigation (including additional factors probably involved in this regulation). Directly silencing of either *MUC1* or *NRG1* by siRNA significantly inhibited NSCLC cell growth and tumour formation in vitro and in vivo, implying that they can be used as potential therapeutic targets for NSCLC. Interestingly, one recent study reveals that MUC1 can activate PD‐L1 expression in NSCLC cells for repression of immune effectors during cancer development.^37^ Another recent study also reports that evodiamine suppresses NSCLC by elevating CD8 + T cells and down‐regulating the MUC1/PD‐L1 axis.^38^ In addition, Pan *et al* recently have developed an anti‐MUC1 antibody‐drug conjugate synthesized by conjugating GSTA neoantigen‐specific 16A with monomethyl auristatin E (MMAE), which displaying potent antitumoural efficacy towards various cancer cells including NSCLC.^39^ A strong correlation between MUC1 and NRG1 expression was also found in NSCLC based on our tissue array data, although the underlying mechanisms of their interaction or their contributions to EIF4G1‐mediated cellular functions still require further investigation.

## CONFLICT OF INTEREST

All the authors declare no conflicts of interest.

## AUTHOR CONTRIBUTION

**Luis Del Valle:** Conceptualization (equal); Formal analysis (equal); Investigation (equal). **Lu Dai:** Conceptualization (equal); Data curation (equal); Formal analysis (equal); Investigation (equal). **Hui‐Yi Lin:** Formal analysis (equal). **Zhen Lin:** Methodology (equal). **Jungang Chen:** Investigation (equal). **Steven Post:** Conceptualization (equal). **Zhiqiang Qin:** Conceptualization (equal); Formal analysis (equal).

## Supporting information

Supplementary Material

## Data Availability

The data sets used and/or analysed during the current study are available from the corresponding author on reasonable request.
