# Supplementary material for: Role of EIF4G1 network in non‐small cell lung cancers (NSCLC) cell survival and disease progression
Source: J Cell Mol Med. 2021 Feb 4;25(6):2795–805. doi: 10.1111/jcmm.16307 (PMC7957206; doi:10.1111/jcmm.16307)
Supplement: Supplementary file 1 — Supplementary Material [file JCMM-25-2795-s001.docx]

| **Position** |  | **Age** |  | **Gender** |  | **Pathology Diagnosis** |  | **TNM** |  | **Grade** |  | **Stage** |  |  |  | **Immunohistochemistry** | | | | |  |  |
| --- | --- | --- | --- | --- | --- | --- | --- | --- | --- | --- | --- | --- | --- | --- | --- | --- | --- | --- | --- | --- | --- | --- |
|  |  |  |  |  |  |  |  |  |  |  |  |  |  | EIF4G1 |  | MUC-1 |  | Heregulin |  | PD-1 |  | PD-L1 |
| A1 |  | 55 |  | Male |  | Squamous Cell Carcinoma |  | T2N0M0 |  | 1 |  | IB |  | ++++ |  | ++++ |  | +++ |  | ++ |  | ++++ |
| A2 |  | 55 |  | Male |  | Cancer adjacent Lung |  | - |  | - |  | - |  | + |  | ++ |  | ++ |  | + |  | + |
| A3 |  | 63 |  | Male |  | Squamous Cell Carcinoma |  | T2N0M0 |  | 1 |  | IB |  | ++++ |  | ++++ |  | ++++ |  | ++ |  | +++ |
| A4 |  | 63 |  | Male |  | Cancer adjacent Lung |  | - |  | - |  | - |  | + |  | ++ |  | ++ |  | + |  | ++ |
| A5 |  | 65 |  | Male |  | Squamous Cell Carcinoma |  | T2N0M0 |  | 1 |  | IB |  | ++ |  | +++ |  | +++ |  | +++ |  | +++ |
| A6 |  | 65 |  | Male |  | Cancer adjacent Lung (pulmonary edema) |  | - |  | - |  | - |  | - |  | ++ |  | ++ |  | + |  | + |
| A7 |  | 72 |  | Male |  | Squamous Cell Carcinoma |  | T2N1M0 |  | 2 |  | IIA |  | +++ |  | ++++ |  | ++++ |  | ++ |  | ++ |
| A8 |  | 72 |  | Male |  | Cancer adjacent Lung |  | - |  | - |  | - |  | - |  | ++ |  | ++ |  | + |  | + |
| A9 |  | 72 |  | Male |  | Squamous Cell Carcinoma |  | T2N0M0 |  | 2 |  | IB |  | ++++ |  | ++++ |  | +++ |  | + |  | ++ |
| A10 |  | 72 |  | Male |  | Cancer adjacent Lung |  | - |  | - |  | - |  | + |  | ++ |  | + |  | + |  | + |
| B1 |  | 56 |  | Male |  | Squamous Cell Carcinoma |  | T2N0M0 |  | 2 |  | IB |  | +++ |  | ++++ |  | +++ |  | - |  | + |
| B2 |  | 56 |  | Male |  | Cancer adjacent Lung |  | - |  | - |  | - |  | + |  | +++ |  | + |  | - |  | + |
| B3 |  | 62 |  | Male |  | Squamous Cell Carcinoma |  | T2N0M0 |  | 2 |  | IB |  | ++++ |  | +++ |  | +++ |  | ++ |  | ++++ |
| B4 |  | 62 |  | Male |  | Cancer adjacent Lung |  | - |  | - |  | - |  | - |  | ++ |  | ++ |  | - |  | + |
| B5 |  | 55 |  | Male |  | Squamous Cell Carcinoma |  | T2N1M0 |  | 2 |  | IIA |  | +++ |  | ++++ |  | ++++ |  | + |  | + |
| B6 |  | 55 |  | Male |  | Cancer adjacent Lung |  | - |  | - |  | - |  | + |  | ++ |  | ++ |  | + |  | + |
| B7 |  | 58 |  | Male |  | Squamous Cell Carcinoma |  | T2N1M0 |  | 2 |  | IIA |  | ++++ |  | +++ |  | +++ |  | ++ |  | +++ |
| B8 |  | 58 |  | Male |  | Cancer adjacent Lung |  | - |  | - |  | - |  | + |  | ++ |  | ++ |  | + |  | + |
| B9 |  | 65 |  | Male |  | Squamous Cell Carcinoma (sparse) |  | T2N0M0 |  | 2 |  | IB |  | ++++ |  | ++++ |  | ++++ |  | +++ |  | +++ |
| B10 |  | 65 |  | Male |  | Cancer adjacent Lung |  | - |  | - |  | - |  | + |  | ++ |  | ++ |  | + |  | + |
| C1 |  | 61 |  | Male |  | Squamous Cell Carcinoma |  | T2N0M0 |  | 2 |  | IB |  | +++ |  | +++ |  | +++ |  | +++ |  | ++ |
| C2 |  | 61 |  | Male |  | Cancer adjacent Lung |  | - |  | - |  | - |  | + |  | ++ |  | + |  | + |  | + |
| C3 |  | 56 |  | Male |  | Squamous Cell Carcinoma |  | T2N0M0 |  | 2 |  | IB |  | ++++ |  | +++ |  | ++++ |  | ++ |  | +++ |
| C4 |  | 56 |  | Male |  | Cancer adjacent Lung |  | - |  | - |  | - |  | + |  | ++ |  | + |  | + |  | ++ |
| C5 |  | 62 |  | Male |  | Squamous Cell Carcinoma |  | T2N1M0 |  | 2 |  | IIA |  | ++++ |  | ++++ |  | ++++ |  | - |  | ++ |
| C6 |  | 62 |  | Male |  | Cancer adjacent Lung |  | - |  | - |  | - |  | + |  | ++ |  | ++ |  | - |  | + |
| C7 |  | 54 |  | Male |  | Squamous Cell Carcinoma (sparse) |  | T2N0M0 |  | 2 |  | IB |  | ++ |  | ++++ |  | +++ |  | ++ |  | +++ |
| C8 |  | 54 |  | Male |  | Cancer adjacent Lung |  | - |  | - |  | - |  | - |  | ++ |  | ++ |  | + |  | + |
| C9 |  | 63 |  | Male |  | Squamous Cell Carcinoma |  | T2N0M0 |  | 3 |  | IB |  | ++++ |  | ++++ |  | +++ |  | + |  | +++ |
| C10 |  | 63 |  | Male |  | Cancer adjacent Lung |  | - |  | - |  | - |  | - |  | ++ |  | + |  | - |  | + |
| D1 |  | 61 |  | Male |  | Squamous Cell Carcinoma |  | T2N0M0 |  | 2 |  | IB |  | ++++ |  | ++++ |  | ++++ |  | + |  | ++ |
| D2 |  | 61 |  | Male |  | Cancer adjacent Lung |  | - |  | - |  | - |  | - |  | ++ |  | ++ |  | - |  | + |
| D3 |  | 59 |  | Male |  | Squamous Cell Carcinoma |  | T2N1M0 |  | 2--3 |  | IIA |  | +++ |  | ++++ |  | ++++ |  | + |  | +++ |
| D4 |  | 59 |  | Male |  | Cancer adjacent Lung |  | - |  | - |  | - |  | - |  | ++ |  | ++ |  | + |  | + |
| D5 |  | 63 |  | Male |  | Squamous Cell Carcinoma |  | T2N1M0 |  | 3 |  | IIA |  | ++++ |  | ++++ |  | ++++ |  | +++ |  | ++++ |
| D6 |  | 63 |  | Male |  | Cancer adjacent Lung |  | - |  | - |  | - |  | + |  | ++ |  | ++ |  | + |  | + |
| D7 |  | 59 |  | Male |  | Squamous Cell Carcinoma |  | T2N3M0 |  | 3 |  | IIIB |  | +++ |  | +++ |  | +++ |  | ++ |  | +++ |
| D8 |  | 59 |  | Male |  | Cancer adjacent Lung |  | - |  | - |  | - |  | + |  | ++ |  | + |  | + |  | ++ |
| D9 |  | 62 |  | Male |  | Squamous Cell Carcinoma |  | T2N1M0 |  | 3 |  | IIA |  | ++ |  | ++ |  | +++ |  | + |  | +++ |
| D10 |  | 62 |  | Male |  | Cancer adjacent Lung (Pulmonary Edema) |  | - |  | - |  | - |  | + |  | ++ |  | +++ |  | + |  | ++ |
| E1 |  | 58 |  | Male |  | Squamous Cell Carcinoma |  | T2N0M0 |  | 3 |  | IB |  | ++++ |  | +++ |  | +++ |  | + |  | +++ |
| E2 |  | 58 |  | Male |  | Cancer adjacent Lung |  | - |  | - |  | - |  | - |  | ++ |  | ++ |  | + |  | + |
| E3 |  | 66 |  | Male |  | Squamous Cell Carcinoma |  | T2N0M0 |  | 3 |  | IB |  | ++++ |  | +++ |  | ++++ |  | + |  | ++ |
| E4 |  | 66 |  | Male |  | Cancer adjacent Lung |  | - |  | - |  | - |  | - |  | ++ |  | + |  | + |  | + |
| E5 |  | 56 |  | Male |  | Squamous Cell Carcinoma |  | T2N1M0 |  | 3 |  | IIA |  | +++ |  | ++++ |  | ++++ |  | + |  | ++ |
| E6 |  | 56 |  | Male |  | Cancer adjacent Lung |  | - |  | - |  | - |  | - |  | ++ |  | + |  | + |  | + |
| E7 |  | 65 |  | Male |  | Squamous Cell Carcinoma |  | T2N0M0 |  | 3 |  | IB |  | ++ |  | ++++ |  | +++ |  | ++ |  | +++ |
| E8 |  | 65 |  | Male |  | Cancer adjacent Lung |  | - |  | - |  | - |  | - |  | ++ |  | ++ |  | + |  | + |
| E9 |  | 54 |  | Male |  | Squamous Cell Carcinoma |  | T2N0M0 |  | 3 |  | IB |  | +++ |  | +++ |  | ++++ |  | + |  | ++ |
| E10 |  | 54 |  | Male |  | Cancer adjacent Lung |  | - |  | - |  | - |  | + |  | ++ |  | ++ |  | + |  | ++ |
| F1 |  | 63 |  | Male |  | Squamous Cell Carcinoma |  | T2N2M0 |  | 3 |  | IIIA |  | +++ |  | +++ |  | +++ |  | + |  | ++++ |
| F2 |  | 63 |  | Male |  | Cancer adjacent Lung |  | - |  | - |  | - |  | + |  | ++ |  | + |  | + |  | + |
| F3 |  | 54 |  | Male |  | Adenosquamous Carcinoma |  | T2N1M0 |  | - |  | IIA |  | ++ |  | +++ |  | +++ |  | ++ |  | +++ |
| F4 |  | 54 |  | Male |  | Cancer adjacent Lung |  | - |  | - |  | - |  | - |  | ++ |  | ++ |  | + |  | + |
| F5 |  | 58 |  | Female |  | Adenocarcinoma |  | T2N1M0 |  | 2 |  | IIA |  | ++++ |  | ++++ |  | +++ |  | +++ |  | +++ |
| F6 |  | 58 |  | Female |  | Cancer adjacent Lung |  | - |  | - |  | - |  | + |  | ++ |  | ++ |  | + |  | + |
| F7 |  | 39 |  | Female |  | Adenocarcinoma |  | T2N0M0 |  | 3 |  | IB |  | +++ |  | +++ |  | +++ |  | +++ |  | ++++ |
| F8 |  | 39 |  | Female |  | Cancer adjacent Lung |  | - |  | - |  | - |  | + |  | ++ |  | + |  | + |  | + |
| F9 |  | 43 |  | Female |  | Invasive Adenocarcinoma |  | T2N0M0 |  | 2 |  | IB |  | ++ |  | ++++ |  | ++++ |  | ++ |  | +++ |
| F10 |  | 43 |  | Female |  | Cancer adjacent Lung |  | - |  | - |  | - |  | - |  | ++ |  | + |  | + |  | + |
| G1 |  | 41 |  | Male |  | Invasive Adenocarcinoma |  | T2N0M0 |  | 2 |  | IB |  | ++ |  | +++ |  | +++ |  | +++ |  | +++ |
| G2 |  | 41 |  | Male |  | Cancer adjacent Lung |  | - |  | - |  | - |  | ++ |  | ++ |  | ++ |  | ++ |  | ++ |
| G3 |  | 69 |  | Male |  | Adenocarcinoma |  | T2N2M0 |  | - |  | IIIA |  | +++ |  | +++ |  | +++ |  | ++++ |  | ++++ |
| G4 |  | 69 |  | Male |  | Cancer adjacent Lung |  | - |  | - |  | - |  | ++ |  | ++ |  | ++ |  | + |  | ++ |
| G5 |  | 64 |  | Female |  | Adenocarcinoma |  | T2N0M0 |  | 2 |  | IB |  | ++++ |  | ++++ |  | ++++ |  | + |  | ++++ |
| G6 |  | 64 |  | Female |  | Cancer adjacent Lung |  | - |  | - |  | - |  | - |  | ++ |  | ++ |  | + |  | + |
| G7 |  | 55 |  | Male |  | Invasive Adenocarcinoma |  | T2N0M0 |  | 2 |  | IB |  | + |  | +++ |  | +++ |  | ++ |  | ++++ |
| G8 |  | 55 |  | Male |  | Cancer adjacent Lung |  | - |  | - |  | - |  | - |  | ++ |  | + |  | + |  | + |
| G9 |  | 57 |  | Male |  | Adenocarcinoma |  | T2N1M0 |  | 2 |  | IIA |  | ++++ |  | ++++ |  | ++++ |  | + |  | +++ |
| G10 |  | 57 |  | Male |  | Cancer adjacent Lung |  | - |  | - |  | - |  | + |  | ++ |  | ++ |  | + |  | + |
| H1 |  | 71 |  | Male |  | Adenocarcinoma |  | T2N1M0 |  | 2--3 |  | IIA |  | ++++ |  | ++++ |  | ++++ |  | +++ |  | +++ |
| H1 |  | 71 |  | Male |  | Cancer adjacent Lung |  | - |  | - |  | - |  | ++ |  | +++ |  | ++ |  | + |  | + |
| H3 |  | 65 |  | Female |  | Mucinous Adenocarcinoma |  | T4N3M0 |  | 3 |  | IIIB |  | + |  | +++ |  | +++ |  | + |  | ++ |
| H4 |  | 65 |  | Female |  | Cancer adjacent Lung |  | - |  | - |  | - |  | - |  | ++ |  | + |  | + |  | + |
| H5 |  | 68 |  | Male |  | Adenocarcinoma |  | T2N0M0 |  | 3 |  | IB |  | +++ |  | ++++ |  | ++++ |  | ++ |  | +++ |

**Supplemental Table 1. The clinical characters and IHC scores for cases from NSCLC tissue arrays.**

| H6 |  | 68 |  | Male |  | Cancer adjacent Lung |  | - |  | - |  | - |  | + |  | ++ |  | ++ |  | + |  | + |
| --- | --- | --- | --- | --- | --- | --- | --- | --- | --- | --- | --- | --- | --- | --- | --- | --- | --- | --- | --- | --- | --- | --- |
| H7 |  | 66 |  | Male |  | Adenocarcinoma |  | T2N2M0 |  | 3 |  | IIIA |  | ++++ |  | +++ |  | ++++ |  | ++ |  | ++++ |
| H8 |  | 66 |  | Male |  | Cancer adjacent Lung |  | - |  | - |  | - |  | + |  | ++ |  | ++ |  | + |  | + |
| H9 |  | 45 |  | Female |  | Adenocarcinoma |  | T2N0M0 |  | 3 |  | IB |  | ++ |  | ++++ |  | ++++ |  | + |  | ++++ |
| H10 |  | 45 |  | Female |  | Cancer adjacent Lung |  | - |  | - |  | - |  | + |  | ++ |  | ++ |  | + |  | + |
| I1 |  | 67 |  | Male |  | Adenocarcinoma |  | T2N0M0 |  | 3 |  | IB |  | ++++ |  | +++ |  | ++++ |  | ++ |  | +++ |
| I2 |  | 67 |  | Male |  | Cancer adjacent Lung |  | - |  | - |  | - |  | + |  | ++ |  | ++ |  | + |  | + |
| I3 |  | 43 |  | Male |  | Invasive Adenocarcinoma |  | T1N0M0 |  | - |  | IA |  | ++ |  | ++++ |  | ++++ |  | +++ |  | ++++ |
| I4 |  | 43 |  | Male |  | Cancer adjacent Lung |  | - |  | - |  | - |  | - |  | ++ |  | + |  | + |  | ++ |
| I5 |  | 52 |  | Male |  | Adenocarcinoma |  | T2N0M0 |  | 3 |  | IB |  | ++++ |  | ++++ |  | ++++ |  | + |  | ++ |
| 6 |  | 52 |  | Male |  | Cancer adjacent Lung |  | - |  | - |  | - |  | + |  | ++ |  | ++ |  | + |  | + |
| I7 |  | 57 |  | Female |  | Adenocarcinoma |  | T2N0M0 |  | 3 |  | IB |  | ++++ |  | ++++ |  | ++++ |  | + |  | ++ |
| I8 |  | 57 |  | Female |  | Cancer adjacent Lung |  | - |  | - |  | - |  | ++ |  | ++ |  | + |  | + |  | + |
| I9 |  | 40 |  | Male |  | Adenocarcinoma |  | T2N0M0 |  | 3 |  | IB |  | ++++ |  | +++ |  | ++++ |  | - |  | +++ |
| I10 |  | 40 |  | Male |  | Cancer adjacent Lung |  | - |  | - |  | - |  | ++ |  | ++ |  | ++ |  | - |  | + |
| J1 |  | 30 |  | Male |  | Lung tissue |  | - |  | - |  | - |  | ++ |  | ++ |  | ++ |  | + |  | + |
| J2 |  | 30 |  | Male |  | Lung tissue |  | - |  | - |  | - |  | + |  | ++ |  | ++ |  | + |  | + |
| J3 |  | 16 |  | Female |  | Lung tissue |  | - |  | - |  | - |  | + |  | ++ |  | ++ |  | + |  | + |
| J4 |  | 16 |  | Female |  | Lung tissue |  | - |  | - |  | - |  | ++ |  | +++ |  | +++ |  | + |  | + |
| J5 |  | 27 |  | Male |  | Lung tissue |  | - |  | - |  | - |  | + |  | ++ |  | ++ |  | + |  | ++ |
| J6 |  | 27 |  | Male |  | Lung tissue |  | - |  | - |  | - |  | + |  | ++ |  | + |  | + |  | ++ |
| J7 |  | 33 |  | Male |  | Lung tissue |  | - |  | - |  | - |  | - |  | ++ |  | ++ |  | + |  | + |
| J8 |  | 33 |  | Male |  | Lung tissue |  | - |  | - |  | - |  | - |  | ++ |  | ++ |  | + |  | + |
| J9 |  | 40 |  | Female |  | Lung tissue |  | - |  | - |  | - |  | + |  | ++ |  | + |  | + |  | ++ |
| J10 |  | 40 |  | Female |  | Lung tissue |  | - |  | - |  | - |  | + |  | +++ |  | ++ |  | + |  | + |

**Supplemental Figure Legends**

**Figure S1. Targeting EIF4G1 does not affect PD-L1 expression from NSCLC cell-lines.** NSCLC cell-lines were transfected with *EIF4G1*-siRNA or control siRNA as described previously, then protein expression was measured by immunoblots.

**Figure S2. Targeting EIF4G1 suppresses NF-κB pathway activity in NSCLC cell-lines.** (**A**) KEGG (Kyoto Encyclopedia of Genes and Genomes) pathway analysis of protein profile changed in EIF4G1 stably knockdown NSCLC cells indicated that the NF-κB signaling pathway was affected by EIF4G1. (**B**) NSCLC cell-lines were transfected with *EIF4G1*-siRNA or control siRNA as described previously, then protein expression was measured by immunoblots. Representative blots from one of two independent experiments were shown.

**Figure S3. Establishment of MUC1 and NRG1 stably knockdown NSCLC cell-lines**. The Dharmacon lentiviral vectors containing 2 shRNA specifically for each targeted gene (KD1 and KD2), and a non-silencing control (NC) shRNA were used to establish MUC1 and/or NRG1 stably knockdown H460 cell-lines, respectively. Protein expression was measured by immunoblots. Representative blots from one of two independent experiments were shown.

**Figure S1**

**
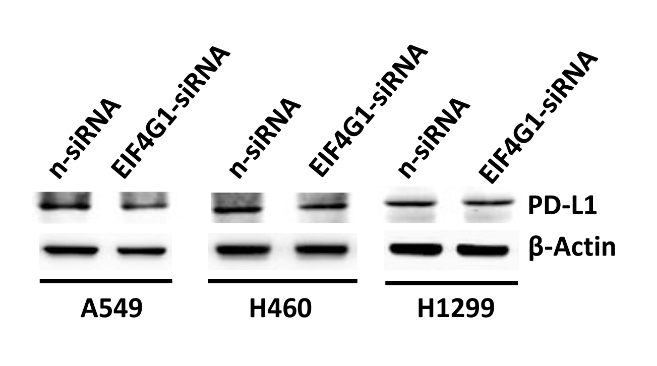
**

**Figure S2**

**
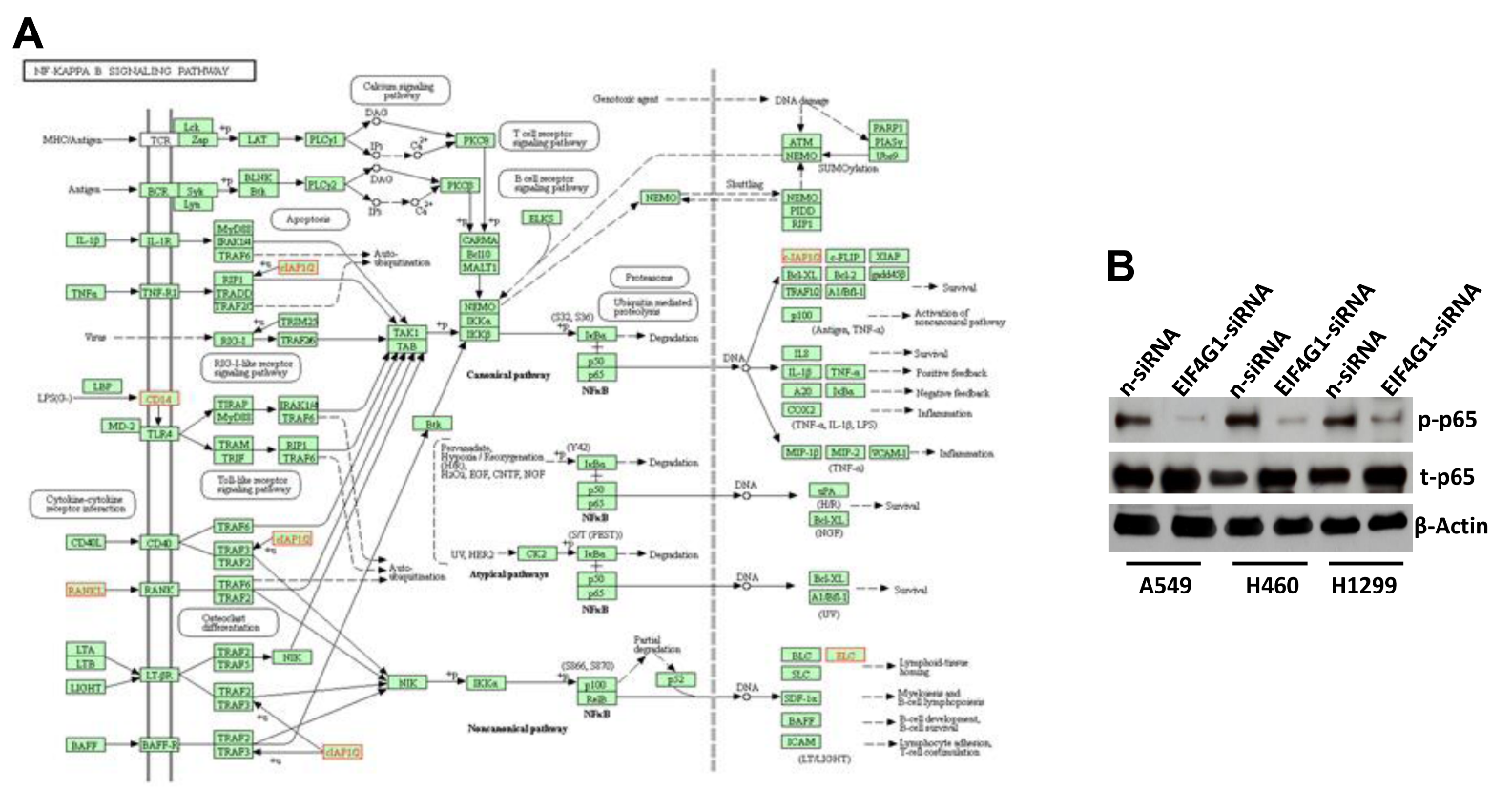
**

**Figure S3**

**
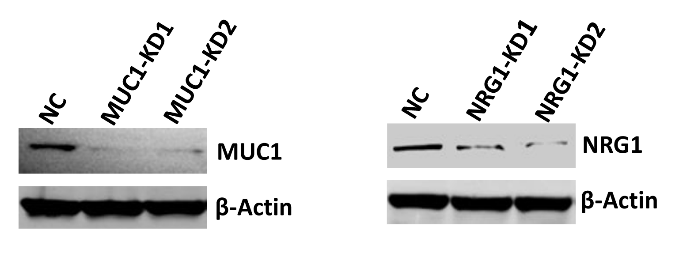
**
